# Segmentectomy-oriented anatomical model for enhanced precision surgery of the left upper lobe

**DOI:** 10.1016/j.xjtc.2023.11.021

**Published:** 2023-12-15

**Authors:** Shunichiro Matsuoka, Takashi Eguchi, Maho Seshimoto, Shuji Mishima, Daisuke Hara, Hirotaka Kumeda, Kentaro Miura, Kazutoshi Hamanaka, Kimihiro Shimizu

**Affiliations:** Division of General Thoracic Surgery, Department of Surgery, Shinshu University School of Medicine, Matsumoto, Nagano, Japan

**Keywords:** left upper segmentectomy, segmental veins, anatomical model, 3-dimensional computed tomography

## Abstract

**Objective:**

To optimize surgical outcomes and minimize complications in complex segmentectomy of the left upper lobe, we investigated the topographical anatomy of the left upper lobe and developed a segmentectomy-oriented anatomical model.

**Methods:**

A state-of-the-art 3-dimensional computed tomography workstation was used to visualize the intersegmental planes and associated veins to categorize the anatomical patterns influencing surgical procedures during left upper lobe segmentectomy. This included the central vein affecting S^1+2^ (apicoposterior segment) segmentectomy, the transverse S^3^ (anterior segment) affecting S^3^ segmentectomy, and other venous branching patterns in 395 patients who underwent thoracic surgery at our institution.

**Results:**

The central vein was observed in 32% of the patients, necessitating access from the interlobar area after segmental artery and bronchus division. Transverse S^3^ incidence was 27%, revealing that only one-third of the patients required complete left upper lobe transection between S^4^ and S^3^ during S^3^ segmentectomy. A significant negative correlation was observed between the presence of transverse S^3^ and the central vein (<10% of patients with the central vein had transverse S^3^ and vice versa). In 6% of patients, the lingular segmental veins partially or entirely drained into the inferior pulmonary vein, potentially causing excessive or insufficient resection during surgery.

**Conclusions:**

This study offers valuable insights into the topographic anatomy of the left upper lobe and presents a segmentectomy-oriented anatomical model for complex segmentectomies. Our approach enables a more precise and individualized surgical planning for patients undergoing segmentectomy based on their unique anatomy, which could thereby lead to improved patient outcomes.

**Figure:**
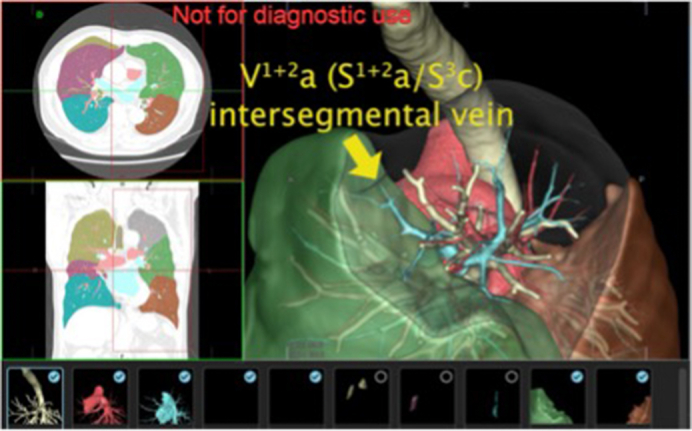
The 3-dimensional planning defines intersegmental planes and veins for segmentectomy.

**Central Message**

The segmentectomy-oriented anatomical model of the left upper lobe using 3-dimensional computed tomography helps identify vital anatomical features, thereby optimizing surgery.

**Perspective**

Using a novel 3-dimensional computed tomography workstation for segmentectomy brings unprecedented precision in identifying intersegmental planes and veins. The insights gained in the present study, such as the negative correlation between transverse S^3^ and the central vein, can lead to more tailored surgeries that reduce complications and enhance patient outcomes.

With the increasing detection of early-stage lung cancer in the older adult population, the use of lung-preserving resections such as wedge resection and segmentectomy has grown globally.^1^^,^^2^ A recent large prospective trial comparing segmentectomy and lobectomy (JCOG0802/WJOG4607L) demonstrated the superiority of segmentectomy over lobectomy for small-sized peripheral non-small lung cancer.^3^ The noninferiority of sublobar resection to lobectomy regarding patient survival, as demonstrated in another large prospective trial comparing sublobar resection (including both segmentectomy and wedge resection) and lobectomy (CALGB140503), further supports the use of sublobar resection, including segmentectomy.^4^

During segmentectomy, proper identification and division of the intersegmental plane are crucial; therefore, the effectiveness with which the corresponding intersegmental vein is managed determines the quality of the segmentectomy. Our group developed an anatomical model using 3-dimensional computed tomography (3D-CT), based on the relationship between the pulmonary vein and bronchus in the right upper lobe (RUL) specifically for successful segmentectomy in the RUL.^5^^,^^6^ Recent studies have explored the complexities of left upper lobe (LUL) bronchovascular anatomy, particularly focusing on the branching patterns of segmental bronchovascular structures. Although these investigations are enhancing the precision of segmentectomies, they have yet to establish a standardized anatomical model.^7^, ^8^, ^9^, ^10^

Simple or typical segmentectomies, defined as segmentectomies that require division of a single, linear intersegmental plane including right S^6^, right basilar (S^7-10^), left upper division (S^1+2^ + S^3^), left lingular (S^4^ + S^5^), left S^6^, or left basilar (S^8-10^), are most frequently performed in the LUL.^11^^,^^12^ The authors of a previous study investigated the lung segmental volume and demonstrated that the left S^1+2^ possessed the largest volume, followed by the right S^3^ and left S^3^.^13^ Given that the LUL includes 2 segments among the top 3 volumetric segments, single complex segmentectomies, such as S^1+2^ or S^3^ segmentectomies, should be considered if oncologically feasible.

Precise identification and division of the intersegmental plane along the corresponding intersegmental vein are crucial for segmentectomy. There are several anatomical challenges for LUL complex segmentectomy, including (1) the left central vein, which is a pulmonary venous branch draining from S^1+2^ and running between the upper division and the lingular segment, which leads to challenges in identification of the intersegmental veins during S^1+2^ segmentectomy; (2) the transverse S^3^ (anterior segment)—the S^3^ that transects the LUL from the anterior to the interlobar hilum requires the transection of the LUL during S^3^ segmentectomy; and (3) other anatomical anomalies of the pulmonary veins, such as an aberrant lingular vein draining into the inferior pulmonary vein (IPV). However, the incidence of these anatomical challenges and their correlations have not yet been reported.

To address the knowledge gaps in the topographical segmental anatomy of the lungs and achieve technical advancements in LUL complex segmentectomy, we investigated the bronchovascular anatomy of the lung using a novel 3D-CT workstation capable of precisely reconstructing intersegmental and intrasegmental veins and creating a segmentectomy-oriented anatomical model.

## Methods

### Reconstruction of 3D-CT

Between May 2017 and December 2021, 413 patients underwent contrast-enhanced CT before thoracic surgery at the Shinshu University Hospital. During this period, we used 2 types of CT scanners: Light Speed VCT Vision (GE Healthcare) until December 2019, and Revolution CT (GE Healthcare) from January 2020 onwards. All contrast-enhanced 3D-CT examinations were performed using 0.63-mm-thick full-resolution scans. The images constructed using Digital Imaging and Communications in Medicine data were transferred to a workstation with the REVORAS (Ziosoft) 3D image-processing software,^14^ and 3D-CT reconstruction images of the pulmonary bronchus and vessels were created using the volume-rendering method. Patients with a history of lung resection in LUL (n = 9), atelectasis in LUL due to the tumor (n = 2), and unevaluable cases due to poor CT images (n = 4) and anatomical abnormalities (n = 3) were excluded. The remaining 395 patients were enrolled in the study, including those with primary lung cancer (n = 273), metastatic lung cancer (n = 57), benign lung tumor (n = 11), mediastinal tumor (n = 11), and other diseases (n = 38). This study was approved by the institutional review board of Shinshu University Hospital (Project ID 4938, Nov 6, 2020), and an opt-out approach for study involvement was used in lieu of obtaining written informed consent.

### Definition of Pulmonary Segments and Pulmonary Veins With Corresponding Drainage Areas

We used the nomenclature of the pulmonary segments and veins in the LUL as described by Nomori and Okada.^15^ The LUL is composed of 4 segments, including the S^1+2^ (apicoposterior segment), S^3^ (anterior segment), S^4^ (lingular superior segment), and S^5^ (lingular inferior segment). There are 3 subsegments (a, b, and c) in the S^1+2^ and S^3^ and 2 subsegments (a and b) in the S^4^ and S^5^. S^1+2^a represents the apical subsegment, S^1+2^b represents the posterior subsegment, and S^1+2^c represents the lateral subsegment of the S^1+2^. S^3^a represents the posterior (lateral) subsegment, S^3^b represents the anterior (medial) subsegment, and S^3^c represents the superior subsegment of the S^3^. S^4^a represents the posterior (lateral) subsegment and S^4^b represents the anterior (medial) subsegment of the S^4^. S^5^a and S^5^b represent the superior and inferior subsegments, respectively, of the S^5^. The pulmonary veins and corresponding drainage areas (vein/drainage segments) were as follows: V^1+2^/S^1+2^, V^3^/S^3^ and S^4^, V^4^/S^4^ and S^5^, and V^5^/S^5^.

Two intersegmental veins run through the intersegmental plane between S^1+2^ and S^3^: V^1+2^a (anterior plane between S^1+2^a and S^3^c) and V^1+2^d (posterior plane between S^1+2^c and S^3^a). Similarly, 2 intersegmental veins run through the intersegmental plane between S^3^ and S^4^: V^3^b (anterior plane between S^3^b and S^4^b). An additional intersegmental vein running through the posterior plane between S^3^a [or S^1+2^c] and S^4^a was not defined in the Nomori and Okada nomenclature. In the study, we have introduced the term V^3^t to define this specific intersegmental vein. One intersegmental vein (V^4^b) runs through the intersegmental plane between S^4^ and S^5^. In general, several intrasegmental veins run inside a lung segment along the intersubsegmental plane between 2 subsegments. The intrasegmental veins running between the 2 subsegments were as follows: V^1+2^b (between S^1+2^a and S^1+2^b), V^1+2^c (between S^1+2^b and S^1+2^c), V^3^a (between S^3^a and S^3^b), V^3^c (between S^3^b and S^3^c), V^4^a (between S^4^a and S^4^b), and V^5^a (between S^5^a and S^5^b).

### Differentiating Pulmonary Veins With a Voronoi Diagram

**Figure 1:**
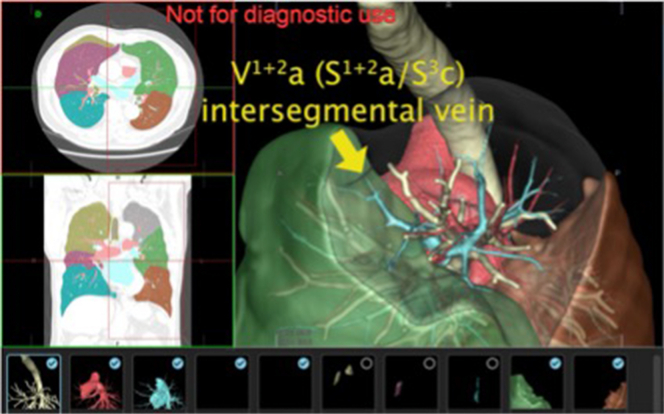
The 3-dimensional planning defines intersegmental planes and veins for segmentectomy.

Using REVORAS, intersegmental or intersubsegmental planes can be determined based on the theory of the Voronoi diagram,^16^ in which an intersegmental plane between segments x (S^x^) and y (S^y^) is located at the midpoint between the 2 peripheral branches from B^x^ and B^y^ (or A^x^ and A^y^). We illustrated the intersegmental or intersubsegmental planes using the segmentectomy planning function of the 3D workstation (Video 1). Pulmonary veins running along the intersegmental or intersubsegmental planes were differentiated based on the aforementioned relationship between the veins and segments (or subsegments) (see Figure 1).

### Anatomical Challenge 1: The Left Central Vein

In our previous study on the RUL, we categorized segmentectomy approaches based on the combination of anterior and central veins.^6^ This framework guided our current investigation on the segmental veins of the LUL, and their anterior and central counterparts.^6^ In the RUL, the central vein is defined as a pulmonary venous branch located in the center of the RUL between the right B^2^ (posterior segmental branch) and B^3^ (anterior segmental branch), running caudally through the interlobar area to the upper lobe bronchus and finally draining into the right superior pulmonary vein (SPV).^5^ By contrast, the anterior vein of the RUL is defined as the pulmonary venous branch located anterior to the RUL bronchus that drains into the SPV.^5^

Although the anterior and central veins have not been defined on the left side, the V^1+2^ of the LUL corresponds closely to the anterior and central veins of the RUL. Similar to that in the right side, the left anterior vein was defined as the pulmonary venous branch draining from the S^1+2^, running anterior to the upper division of the bronchus (B^1+2^ + B^3^), and finally draining into the left SPV. In contrast, the left central vein was defined as the pulmonary venous branch draining from the S^1+2^, initially running between B^1+2^ and B^3^, subsequently running around B^3^ posteriorly, and finally running between B^3^ and the lingular bronchus (B^4+5^) and draining into the left SPV.

We classified V^1+2^ into 3 subtypes based on the combinations of the anterior and central veins: anterior type (no central vein), anterior with central type (both anterior and central veins), and central type (no anterior vein). The anterior with central type was further divided into the anterior “a” with central type and the anterior “ab” with central type. In the “a” type, the V^1+2^a (inter-segmental vein between S^1+2^ and S^3^) drains into the anterior vein and the V^1+2^b (intra-segmental vein of S^1+2^) drains into the central vein. In the “ab” type, both the V^1+2^a and V^1+2^b drain into the anterior vein. The incidence of each subtype was also determined.

### Anatomical Challenge 2: The Transverse S^3^

During left S^3^ segmentectomy, some surgeons prefer to transect the LUL between the upper division (S^1+2^ + S^3^) and lingular segment (S^4^ + S^5^) and then divide the intersegmental plane between S^1+2^ and S^3^ to complete S^3^ segmentectomy. This maneuver, which involves transection of the LUL, is based on the belief that the S^3^ is located in the middle of the LUL and crosses from the anterior hilum to the interlobar area. However, in some cases, the S^3^ is only located in the anterior area of the upper division, and the posterior area of the upper division only comprises the S^1+2^. We defined the S^3^ that transverses from the anterior hilum to the interlobar area as the transverse type and the S^3^ that does not transverse as the nontransverse type. The incidence of transverse and nontransverse S^3^ was investigated. The χ^2^ test was performed using SPSS version 27 (SPSS Inc) to analyze the relationship between S^3^ transverse and the central vein.

### Anatomical Challenge 3: The Variety of Inter- or Intrasegmental Veins Draining From the S^3^ Segment

During S^3^ segmentectomy, the intersegmental veins between S^3^ and S^1+2^ (eg, V^1+2^a) and between S^3^ and S^4^ (eg, V^3^b) will be divided or preserved, depending on the unique anatomical and oncologic conditions. However, the intrasegmental vein of S^3^ (V^3^c), which drains into the SPV, the anterior vein, or V^3^b, should be divided during S^3^ segmentectomy. Therefore, knowledge of the drainage pattern of the V^3^c before S^3^ segmentectomy is crucial for developing an appropriate segmentectomy plan for S^3^ segmentectomy. We investigated the drainage pattern of the V^3^c and classified it according to the drainage veins of the V^3^c.

### Anatomical Challenge 4: The Variety of Lingular Segmental Vein (V^4+5^)

During lingulectomy (S^4^+S^5^ segmentectomy), the V^4+5^ drainage vein is divided. In most cases, the procedure is simple. However, a potentially challenging drainage pattern of the V^4+5^ may be encountered during lingulectomy, including multiple stems of the V^4+5^ and abnormal V^4+5^ drainage into the IPV. The drainage patterns were investigated in this study.

## Results

### Incidence of Central Vein

**Figure 2:**
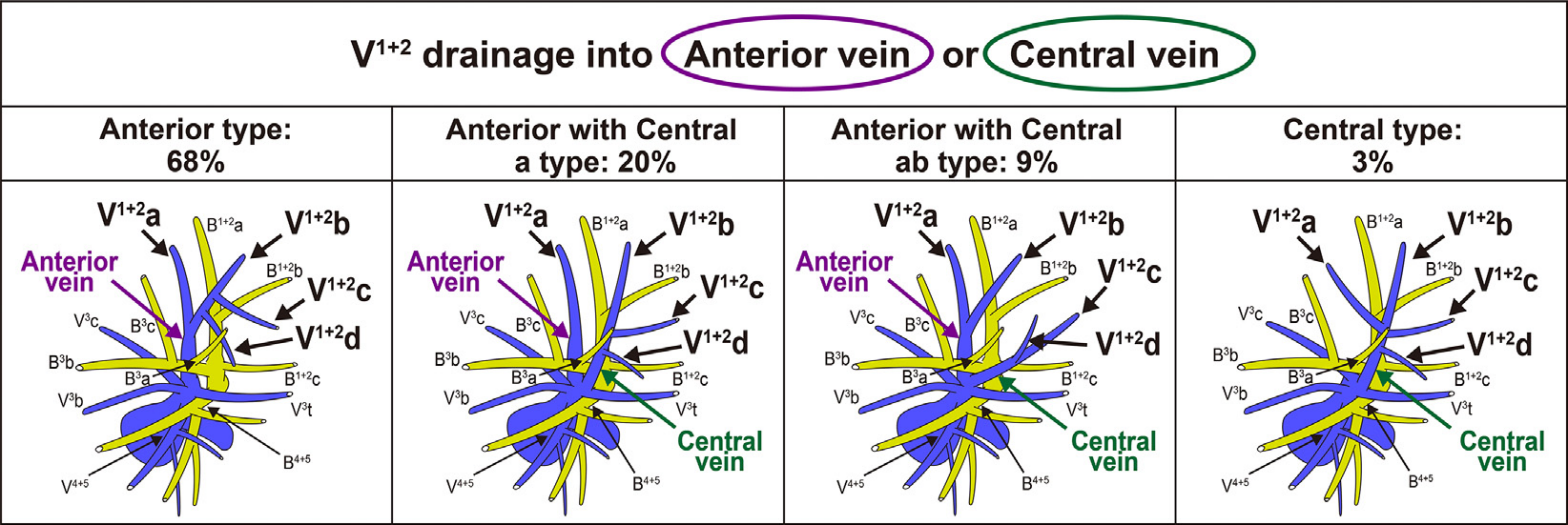
V^1+2^ classification according to the combination of the anterior vein and central vein.

**Figure E1:**
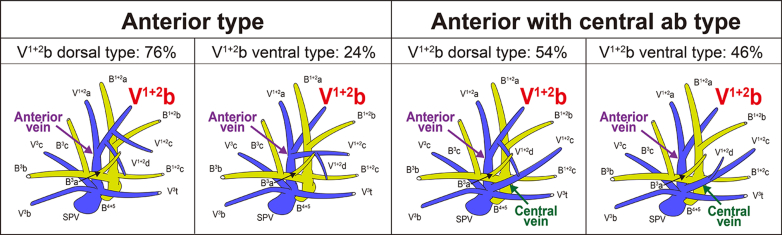
Subclassification was based on V^1+2^b running through the dorsal or ventral side of B^1+2^ in the anterior type, and the anterior with central ab type.

**Figure E2:**
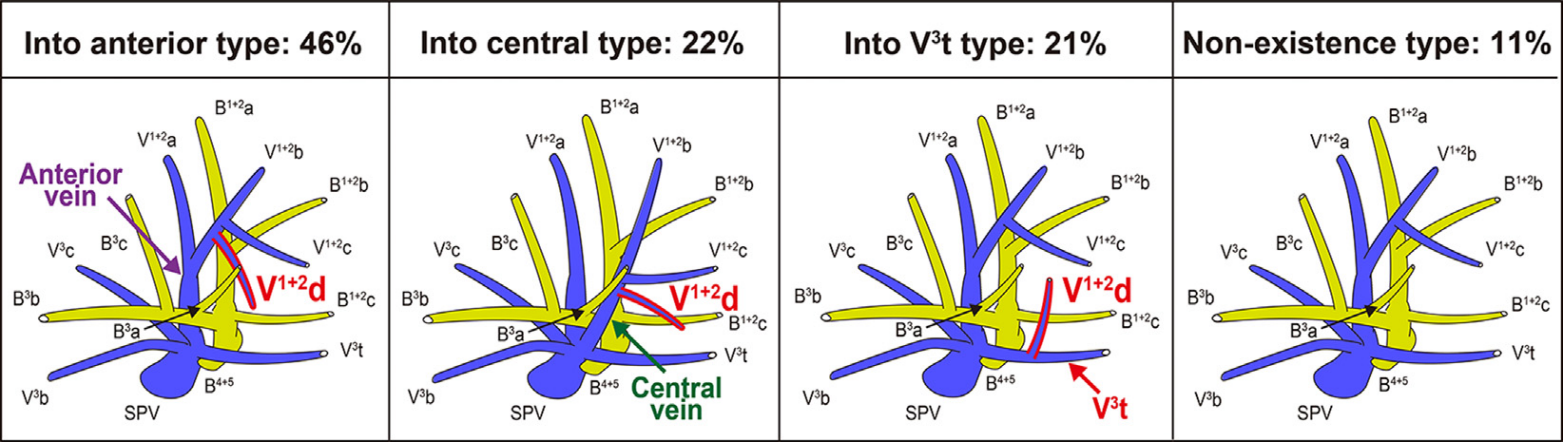
Drainage patterns of V^1+2^d running between S^1+2^c and S^3^a. *SPV*, Superior pulmonary vein.

The central vein was observed in 126 patients (32%), indicating that during S^1+2^ segmentectomy, the intersegmental vein between S^1+2^ and S^3^ would be identified and exposed in one-third of the patients only after dividing the artery and bronchus. The most frequent V^1+2^ subtype based on the combination of the central and anterior veins was the anterior type (68%), followed by the anterior “a” with central (20%), the anterior “ab” with central (9%), and central (3%) types (Figure 2). Further branching patterns of the V^1+2^, based on V^1+2^b and V^1+2^d are illustrated in Figures E1 and E2, respectively.

### Incidence of the Transverse S^3^ and Its Correlation With the Central Vein

**Figure 3:**
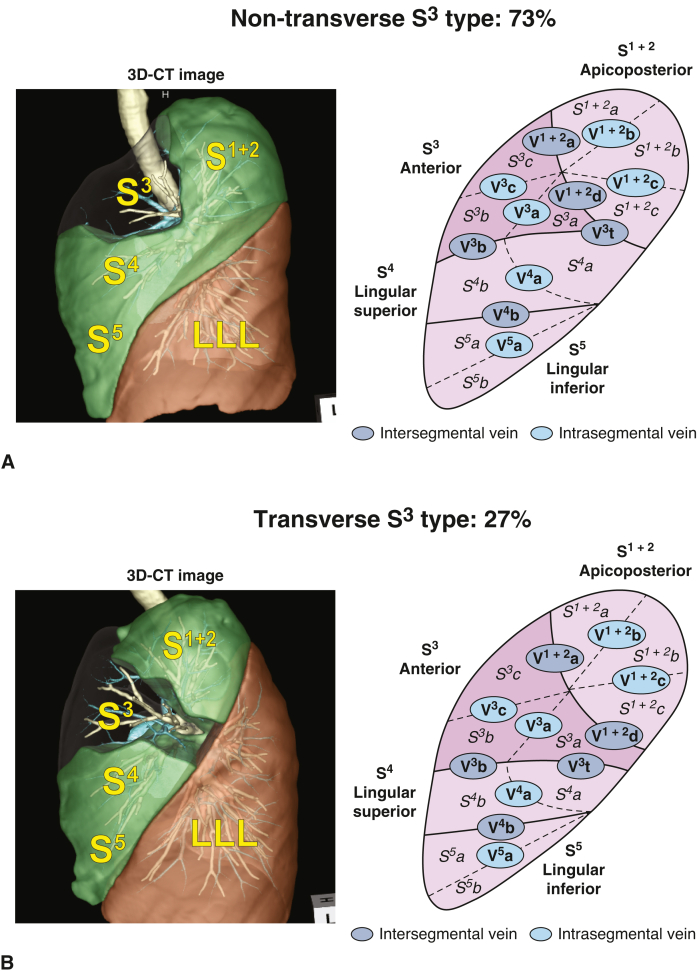
Representative images of (B) transverse and (A) nontransverse S^3^ types with schematic depiction of the intra- and intersegmental veins. *3D-CT*, Three-dimensional computed tomography; *LLL*, left lower lobe.

A transverse pattern of S^3^ was observed in 108 patients (27%) and a nontransverse pattern in 287 patients (73%) (Figure 3). This result suggests that during S^3^ segmentectomy, less than one-third of patients require transection of the LUL. The potential correlation between the presence of the transverse S^3^ and that of the central vein was also examined, as both significantly affected LUL segmentectomy. Among the 108 patients with transverse S^3^, the central vein was only observed in 4 patients (4%). Conversely, among 126 patients with central vein involvement, the transverse S^3^ was only observed in four patients (3%). Therefore, we identified a significant negative correlation between transverse S^3^ and the central vein (*P* < .001) (Table 1).

**Table 1:** Correlation between the transverse S^3^ type and the central vein

S^3^ type	Central vein		*P* value
	Present (n = 126)	Absent (n = 269)	
Transverse (n = 108)	4 (3)	104 (39)	<.001
Nontransverse (n = 287)	122 (97)	165 (61)	

Data are shown as number (%).

### V^3^ Classification Based on the V^3^c Drainage Pattern

**Figure 4:**
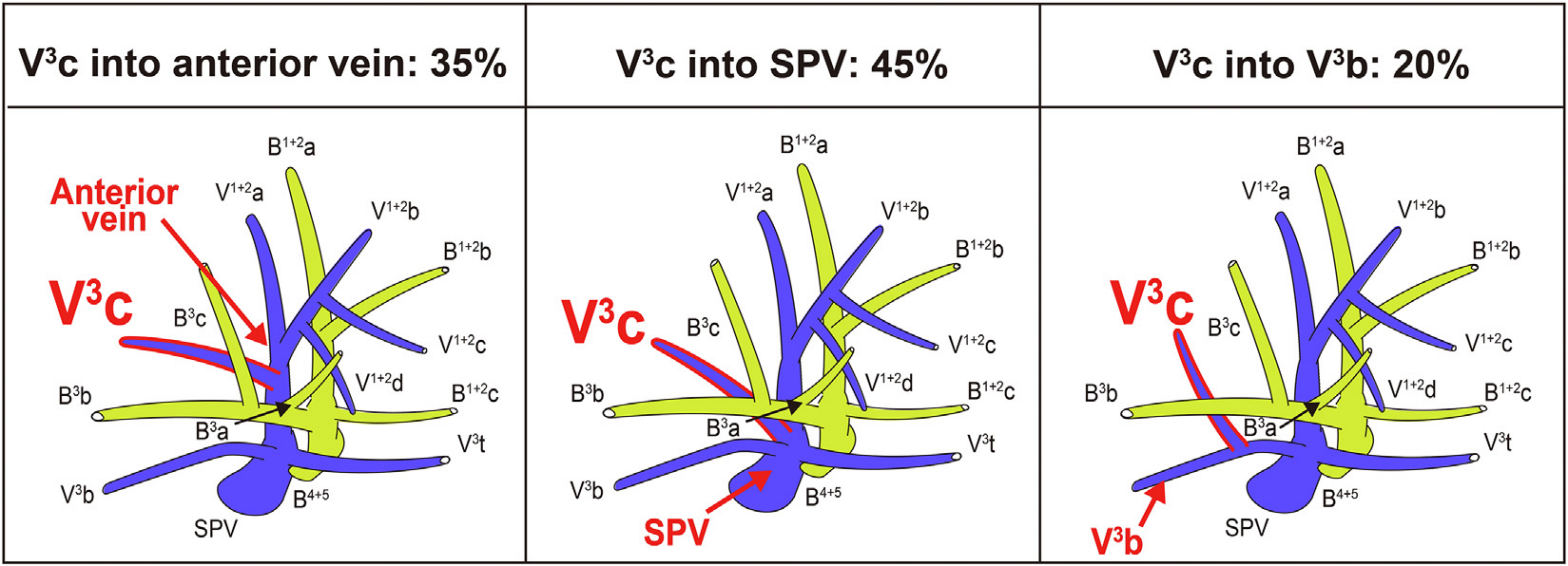
V^3^ classification according to the drainage site of V^3^c. *SPV*, Superior pulmonary vein.

V^3^, the drainage vein from S^3^, was classified into 3 types based on V^3^c (intrasegmental vein of S^3^): (1) V^3^c into the anterior vein, (2) V^3^c into the SPV, and (3) V^3^c into V^3^b. The V^3^a (posterior intrasegmental vein) was excluded from this classification because of numerous drainage patterns and nonexistence in one-third of the cases (Table E1). The most common type was V^3^c into the SPV (45%), followed by V^3^c into the anterior vein (35%), and V^3^c into V^3^b (20%) (Figure 4).

### V^4+5^ Classification Based on the Number of Stems and the Presence of Draining Into the IPV

**Figure 5:**
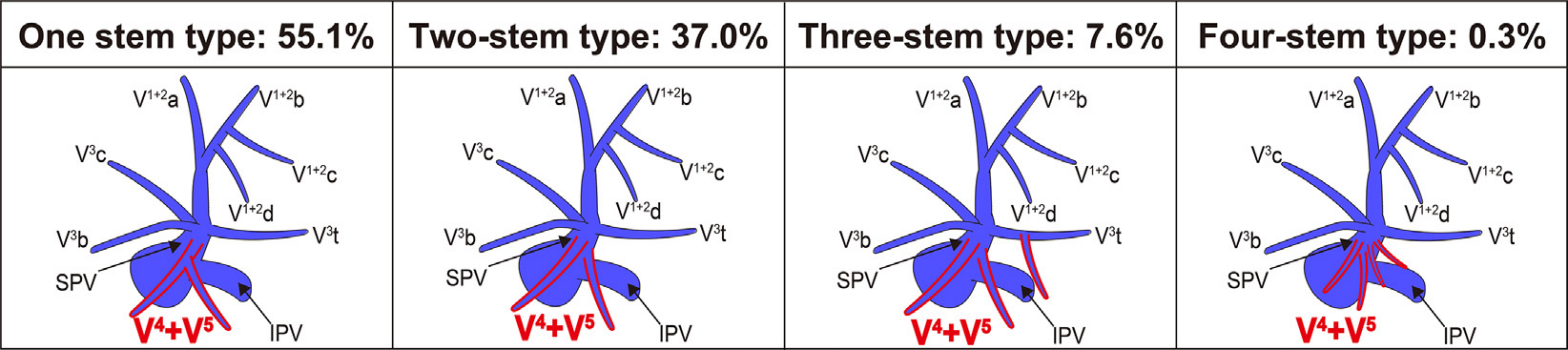
V^4+5^ classification according to the number of V^4+5^ stems. *SPV*, Superior pulmonary vein; *IPV*, inferior pulmonary vein.

**Figure 6:**
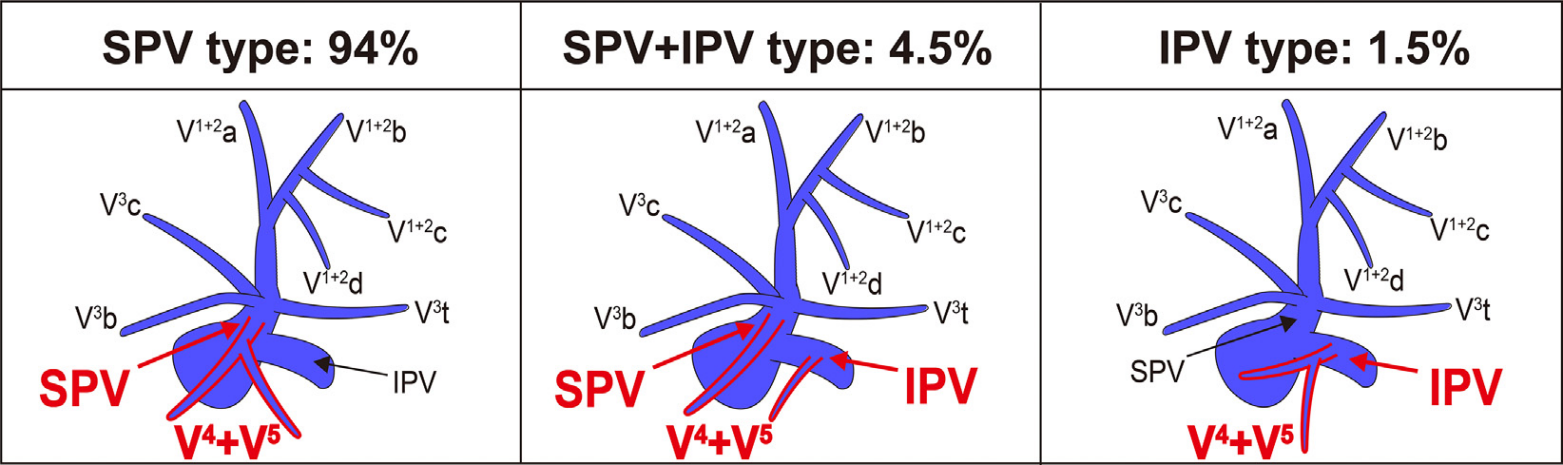
V^4+5^ classification according to the drainage site of V^4+5^ into the superior pulmonary vein and inferior pulmonary vein. *SPV*, Superior pulmonary vein; *IPV*, inferior pulmonary vein.

Figure 5 shows the V^4+5^ classification based on the number of V^4+5^ stems, and detailed drainage patterns are provided in Table E2. The one-stem type accounted for 55.1% of the cases, followed by the 2-stem (37%), 3-stem (7.6%), and 4-stem (0.3%) types. In addition, the branching pattern of V^4+5^ was classified into 3 types according to whether V^4+5^ drains into IPV (Figure 6): the most common were SPV (94%), followed by SPV + IPV (4.5%), and IPV (1.5%) types.

## Discussion

In this study, we delved into the bronchovascular anatomy of the LUL by focusing on its segmental topographical anatomy. We developed a segmentectomy-oriented anatomical model with several strengths and novelties: (1) It allows for automated and precise identification of inter-segmental planes, including intersubsegmental and intrasegmental planes, based on the Voronoi diagram theory. This, in turn, enables accurate identification and classification of the corresponding veins. (2) Through this model, novel segmentectomy-oriented anatomical features, such as a negative relationship between the transverse S^3^ and central vein, were discovered. These findings promote the development of streamlined and tailored approaches for precision segmentectomy in the LUL.

Boyden^17^ and Yamashita^18^ described the segmental anatomy of the lung before the advent of CT, which has been widely accepted among thoracic surgeons. In these studies, segmental distribution, including the location of the intersegmental planes, was based on findings obtained by injecting a colored gelatin solution into the segmental bronchus and vessels using the lungs of human cadavers.^18^ The drawbacks of traditional anatomical methodologies include the requirement for cadavers and their time-consuming nature. Recently, advancements in 3D-CT technology have enabled the peripheral visualization of bronchovascular structures, contributing to improvements in anatomical classification, preoperative simulation, and intraoperative navigation.^19^^,^^20^ Early 3D-CT, however, was unsuitable for identifying the intersegmental planes; therefore, identification of the corresponding intersegmental vein depended solely on the shape and pattern of adjacent segmental bronchovascular structures that were shown on 3D images.^5^^,^^21^^,^^22^ However, recent technical advancements in 3D-CT have enabled precise identification of the intersegmental and intersubsegmental planes. A novel 3D-CT software, “REVORAS,” can draw peripheral bronchovascular structures more accurately than previous 3D-CT software using artificial intelligence and revised algorithms. Based on accurate peripheral bronchial and pulmonary arterial structures, virtual intersegmental and intersubsegmental planes can be precisely depicted using the Voronoi diagram theory. Through this approach, we observed an unnamed intersegmental vein (which we have defined V^3^t) in 375 patients (98%) that runs between S^3^a (or S^1+2^c) and S^4^a, as per the Nomori and Okada nomenclature. This finding deviates from previous reports^7^, ^8^, ^9^, ^10^ and enriches our anatomical comprehension of the posterior plane between the left upper division and lingular segments. Therefore, this function is useful not only for preoperative simulation and intraoperative navigation but also for the precise identification of intersegmental and intersubsegmental (intrasegmental) veins running along the corresponding planes.

Previous studies have examined the pattern of segmental veins in the LUL but have often provided ambiguous definitions of the segmental, anterior, and central veins. For instance, Zhang and colleagues^7^ categorized patterns based on a combination of anterior and central veins and found a different distribution than our study. This discrepancy may arise from potential confusion between V^1+2^d and V^3^t. In our study, we have clarified these definitions and established a segmentectomy-oriented anatomical model, which gives rise to the following clinical implications.

**Figure E3:**
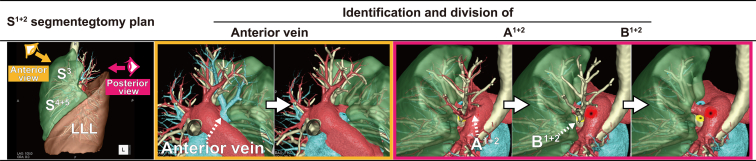
Representative case displaying S^1+2^ segmentectomy plan for a patient with the central vein from the cranial and posterior views using 3-dimensional computed tomography imaging. *LLL*, Left lower lobe.

**Figure E4:**
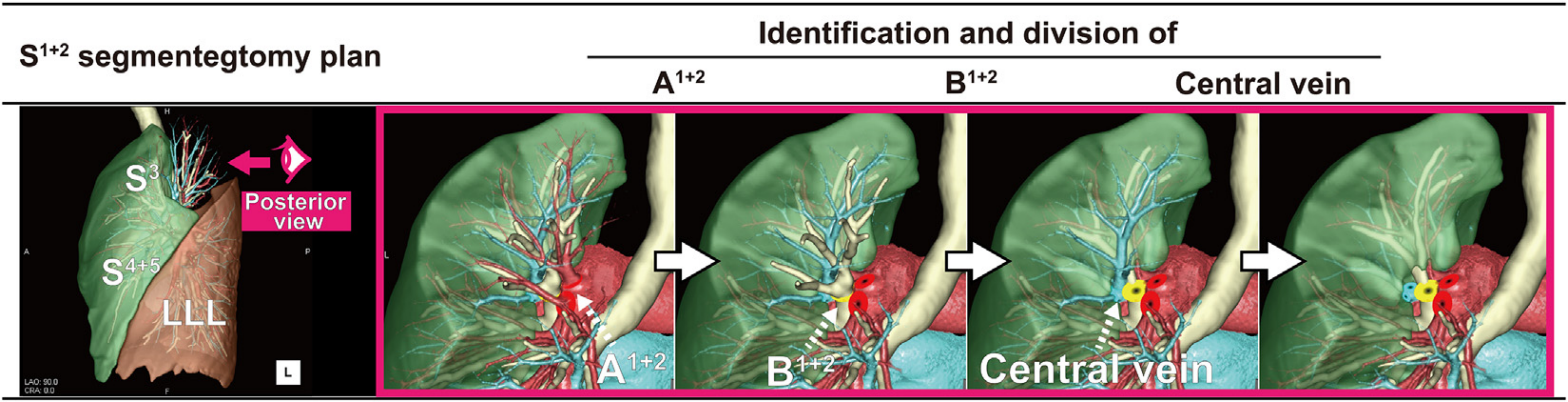
Representative case of the S^1+2^ segmentectomy plan for a patient with the central vein showing 3D-CT cranial and posterior views. The central vein was only identified in the interlobar area after dividing the segmental artery and bronchus. *3D-CT*, Three-dimensional computed tomography; *LLL*, left lower lobe.

In this study, we focused on the anatomically specific features directly related to left S^1+2^ and S^3^ segmentectomies, which are the most commonly performed single segmentectomies.^11^^,^^23^^,^^24^ Intraoperative identification of the intersegmental veins and dissection along them are key procedures during lung segmentectomy. Specifically, during S^1+2^ segmentectomy, the intersegmental veins between S^1+2^ and S^3^ (V^1+2^a and V^1+2^d) should be identified intraoperatively. In the case of anterior V^1+2^, which is the most popular subtype of V^1+2^ (68%), the intersegmental veins can be easily accessed from the anterior hilum, which means that we can identify the intersegmental plane at the beginning of surgery (Figure E3). However, in the case of the central vein (32%), at least 1 of the 2 intersegmental veins could not be identified from the anterior hilum. Instead, access to these veins was only possible from the interlobar area after dividing the segmental artery and bronchus (Figure E4). Therefore, in patients undergoing left S^1+2^ segmentectomy, the presence of a central vein should be checked preoperatively because it significantly affects the surgical procedure.

We also investigated the transverse S^3^ and central veins and demonstrated their incidence and mutually exclusive relationships. During S^3^ segmentectomy, transection of the LUL is not required, and segmentectomy can be completed only by anterior dissection in cases of non-transverse S^3^, which is more than twice as common as transverse S^3^ (nontransverse vs transverse, 73% vs 27%). We prefer to avoid transection of the LUL during S^3^ segmentectomy in cases of nontransverse S^3^, which leads to reduction of unnecessary stapling and potential removal of the lung parenchyma of the adjacent segment.

In this study, we used a novel 3D-CT workstation that enabled evaluation of the shape and distribution of each segment, including presence of the transverse S^3^. However, most conventional 3D-CT workstations do not possess this function, which is one of the limitations of this study regarding its application in clinical practice. Our study found a strong negative correlation between the transverse S^3^ and central vein. This finding helps predict the presence of transverse S^3^, since the possibility of transverse S^3^ is low in patients with a central vein, which can be detected by conventional 3D-CT images.

**Figure E5:**
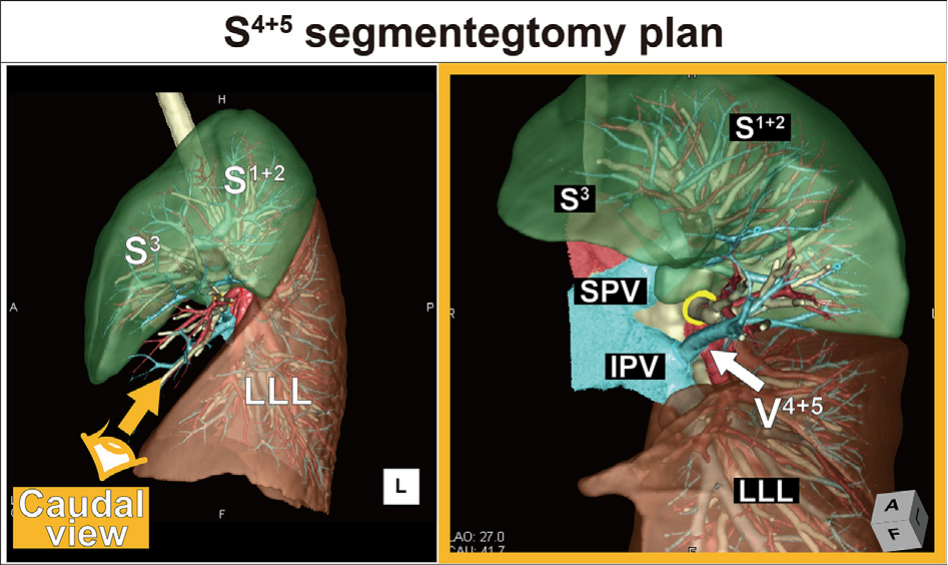
Representative case displaying a patient with V^4+5^ draining into the inferior pulmonary vein. *LLL*, Left lower lobe; *SPV*, superior pulmonary vein; *IPV*, inferior pulmonary vein.

We analyzed the V^4+5^ classification according to the number of V^4+5^ stems by referring to analysis of the right middle lobe^22^ and divided them into 1 to 4 stem types. Furthermore, our findings of another V^4+5^ classification can be used not only in segmentectomy, but also in lobectomy. We found an aberrant lingular vein draining into the IPV in 6% of patients (Figure E5). This anomaly may cause significant issues during both left upper and lower lobectomies, including congestion of the lingular segment after the left lower lobectomy, bleeding from the interlobar area during the left upper lobectomy, and potential excessive division of both the SPV and IPV based on an intraoperative misunderstanding of the pulmonary veins. To avoid possible malpractice during surgery, we should carefully evaluate 3D-CT images preoperatively.

In the accompanying video, we highlighted 3 pivotal aspects of LUL segmentectomy bearing clinical relevance that require further elucidation. First, the frequency of LUL transection in S^3^ segmentectomy is crucial for preoperative planning and can influence surgical outcomes. Second, understanding the prevalence of the central vein in the LUL is vital as it dictates the decision regarding surgical approach and the difficulty of identifying inter-segmental planes. Finally, recognizing common aberrant draining patterns of the lingular vein (V^4+5^) is essential for avoiding complications such as bleeding or inadvertent vessel ligation. These elements collectively serve to enhance surgical planning, intraoperative navigation, and ultimately, patient outcomes, and their further exploration may offer valuable insights that can be integrated into a more individualized and effective surgical strategy for LUL segmentectomy.

This study had several limitations, including its retrospective nature, relatively small patient cohort, and single-institutional design that included only Japanese patients, that could have affected study outcomes. In addition, we analyzed the major inter- and intrasegmental veins in the LUL and did not include the following in our current analysis: small venous branches, bronchial branching patterns, and arterial branching patterns. Furthermore, the usefulness of this model for LUL segmentectomy has not been evaluated.

In conclusion, in this study, we developed a segmentectomy-oriented anatomical model of the LUL using a novel 3D-CT workstation to accurately identify the inter-segmental planes and corresponding veins. Our findings provide valuable insights into the topographical anatomy for complex LUL segmentectomies. Moreover, we believe that our approach could lead to more precise and individualized surgery for patients undergoing segmentectomy, tailored to each patient's unique anatomy.

## Conflict of Interest Statement

The authors reported no conflicts of interest.

The *Journal* policy requires editors and reviewers to disclose conflicts of interest and to decline handling or reviewing manuscripts for which they may have a conflict of interest. The editors and reviewers of this article have no conflicts of interest.

## Figures and Tables

**Table E1:** Drainage pattern of V^3^a, the intra-segmental vein between S^3^a and S^3^b

Drainage site of V^3^a	n = 375
No existence	130 (32.9)
V^3^t	127 (32.2)
V^3^b	117 (29.6)
Central vein	14 (3.5)
Anterior vein	3 (0.8)
V^3^c	2 (0.5)
V^1+2^d	2 (0.5)

Values are presented as n (%).

**Table E2:** Details of the drainage patterns of V^4+5^ according to V^4+5^ stem classification

Classification of lingular vein							
One stem type (n = 218) (55.11%)∗		Two-stem type (n = 146) (37.0%)∗		Three-stem type (n = 30) (7.6%)∗		Four-stem type (n = 1) (0.3%)∗	
SPV	205 (94.0)	SPV + SPV	61 (41.8)	SPV + SPV + V^3^t	10 (33.3)	SPV + SPV + SPV + SPV	1 (100)
Common stem with V^3^	8 (3.7)	SPV + V^3^t	37 (25.3)	SPV + V^3^b + V^3^t	6 (19.8)		
IPV	5 (2.3)	SPV + V^3^b	25 (17.1)	SPV + SPV + SPV	3 (9.9)		
		SPV + IPV	10 (6.8)	SPV + V^3^t + IPV	3 (9.9)		
		SPV + central vein	6 (4.2)	SPV + SPV + V^3^b	3 (9.9)		
		SPV + V^8^	2 (1.4)	SPV + SPV + IPV	2 (6.6)		
		V^3^b + V^3^b	1 (0.7)	SPV + V^3^a + V^3^t	1 (3.3)		
		V^3^t + V^3^t	1 (0.7)	SPV + V^3^b + IPV	1 (3.3)		
		V^3^t + IPV	1 (0.7)	SPV + V^3^b + V^3^b	1 (3.3)		
		Central vein + IPV	1 (0.7)				
		IPV + IPV	1 (0.7)				

Values are presented as n (%). *SPV*, Superior pulmonary vein; *IPV*, inferior pulmonary vein.

∗ Percentages in parentheses indicate the proportion according to the classification of the lingular vein.

## Supplementary Data

Video 1 This video demonstrates the application of our 3D-CT workstation, REVORAS, in precisely identifying intersegmental planes and their corresponding veins in the left upper lobe. The video further elucidates how this technology will contribute to resolving key clinical questions in segmentectomy and offers a practical overview of the intricate anatomical features crucial for successful segmentectomy procedures. Video available at: https://www.jtcvs.org/article/S2666-2507(23)00475-3/fulltext.
