# Supplementary figures and images for: Segmentectomy-oriented anatomical model for enhanced precision surgery of the left upper lobe
Source: JTCVS Tech. 2023 Dec 15;23:92–103. doi: 10.1016/j.xjtc.2023.11.021 (PMC10859669; doi:10.1016/j.xjtc.2023.11.021)

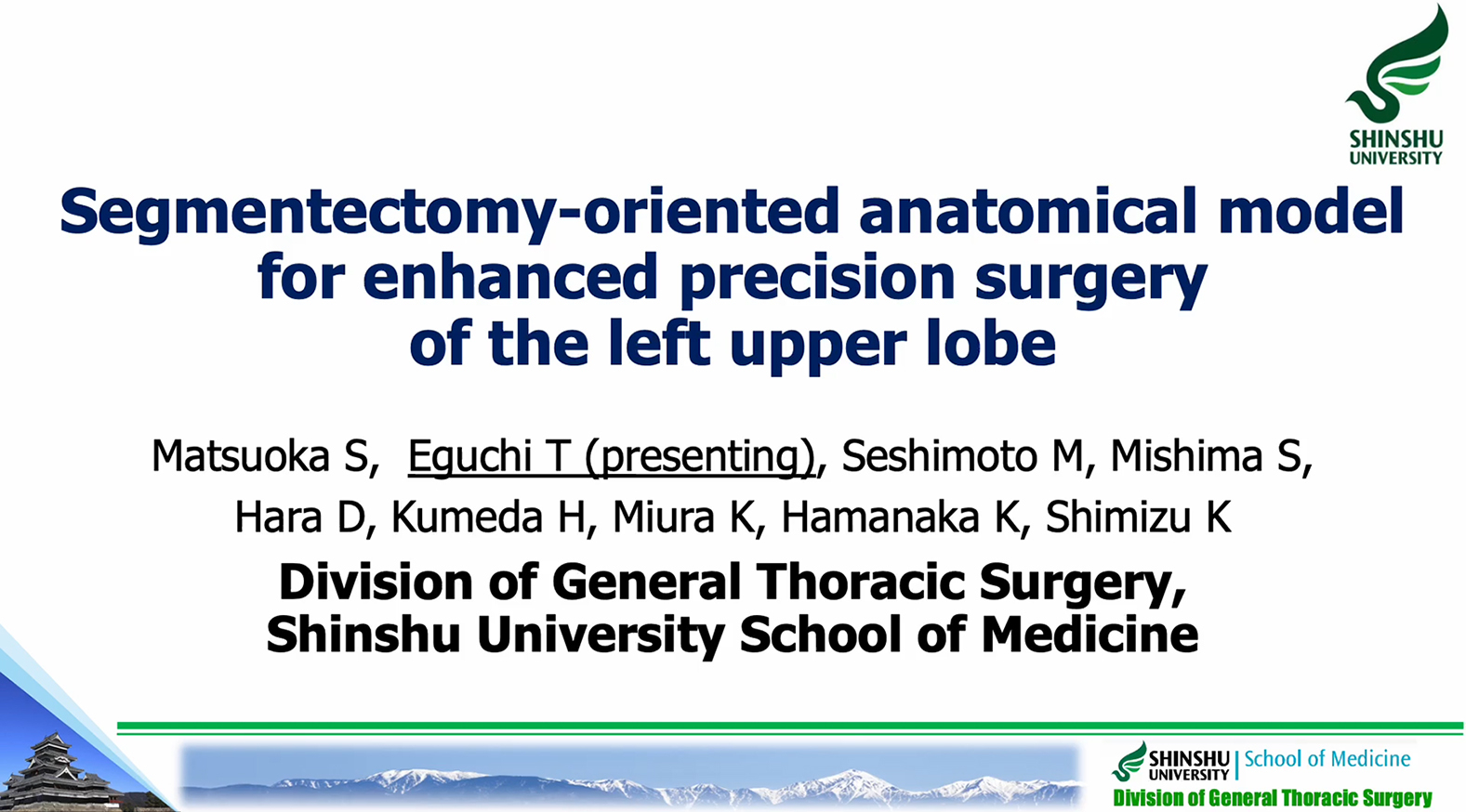

Supplement: Video 1 — This video demonstrates the application of our 3D-CT workstation, REVORAS, in precisely identifying intersegmental planes and their corresponding veins in the left upper lobe. The video further elucidates how this technology will contribute to resolving key clinical questions in segmentectomy and offers a practical overview of the intricate anatomical features crucial for successful segmentectomy procedures. Video available at: https://www.jtcvs.org/article/S2666-2507(23)00475-3/fulltext. [file fx2.jpg]
